# Rare Presentation of Cecocolic Intussusception From a Colonic Lipoma: A Case Report

**DOI:** 10.7759/cureus.89696

**Published:** 2025-08-09

**Authors:** Mohammed A Alsuhaimi, Mohmmed A AlHewishel, May S AlKhaldi, Abdurahman S AlSumaihi, Hadi H AlDossri, Eman F AlSaleh

**Affiliations:** 1 General Surgery, Dammam Medical Complex, Dammam, SAU; 2 Pathology and Laboratory Medicine, Regional Lab, Dammam, SAU

**Keywords:** adult intussusception, bariatric surgery, colo-colic intussusception, colonic lipoma, ct imaging, gastrointestinal lipoma, limited right hemicolectomy

## Abstract

Adult intussusception is an uncommon cause of bowel obstruction, accounting for less than 5% of cases, and is often linked to a pathological lead point. Among benign etiologies, colonic lipomas are rare but recognized causes. We report a case of a 52-year-old male patient who presented with an atypically prolonged one-week history of intermittent abdominal pain and nausea, without signs of overt obstruction. Physical examination revealed abdominal tenderness and hypoactive bowel sounds. Contrast-enhanced computed tomography (CT) demonstrated a rare cecocolic intussusception with a fat-density mass in the transverse colon, suggestive of a colonic lipoma.

Exploratory laparotomy confirmed a large submucosal lipoma acting as the lead point. Given the patient’s age and the imaging ambiguity regarding malignancy, a limited right hemicolectomy was performed. Histopathology confirmed a benign submucosal lipoma. Postoperative recovery was uneventful, and the patient remained symptom-free at three-month follow-up. This case highlights an atypical, subacute presentation of adult intussusception caused by a colonic lipoma. It underscores the diagnostic utility of CT imaging and the rationale for an oncologically safe resection in uncertain cases, even with benign imaging features.

## Introduction

Intussusception in adults is a rare clinical entity, accounting for approximately 1-5% of all cases of bowel obstruction and only 5% of all intussusceptions overall [1]. In contrast to pediatric cases, which are usually idiopathic, adult intussusception is typically secondary to a pathological lead point, either benign (e.g., lipoma, polyp) or malignant (e.g., adenocarcinoma, lymphoma) [2]. Among benign causes, gastrointestinal lipomas are infrequent yet well-recognized lead points. These submucosal tumors, composed of mature adipose tissue, are often incidental findings but may become symptomatic when larger than 4 cm, leading to complications such as obstruction or intussusception [3].

Cecocolic intussusception, where the cecum telescopes into the ascending or transverse colon, is particularly uncommon in adults, comprising a small fraction of all colonic intussusceptions [4]. In a systematic review, colonic intussusceptions accounted for about 20-25% of adult cases, with cecocolic types being among the least reported [5]. Accurate preoperative diagnosis is often difficult due to vague and intermittent symptoms such as abdominal pain, nausea, and altered bowel habits. CT imaging remains the gold standard for diagnosis, with hallmark features such as the “target sign” or “bowel-within-bowel” configuration [6].

This case highlights a rare cecocolic intussusception in a patient with a prior history of mini-gastric bypass, an additional anatomical factor that may complicate the clinical presentation and surgical approach. The lead point was identified as a large submucosal lipoma in the transverse colon. This report underscores the diagnostic challenges, the value of cross-sectional imaging, and the need for a high index of suspicion in adult patients presenting with subacute abdominal symptoms.

## Case presentation

A 52-year-old male patient, previously healthy with no significant past medical history, presented to the emergency department with a one-week history of intermittent, crampy abdominal pain. Initially, the pain was mild, localized to the periumbilical and left upper quadrant regions, and occurred once or twice daily without alarming features. However, over the following four days, the episodes became more frequent and intense, disturbing his sleep and impairing his oral intake. By the sixth day, the pain shifted to involve both the right and left upper quadrants and was described as constant and moderate in severity. Associated symptoms included anorexia, nausea, and bloating. He denied vomiting, hematemesis, weight loss, changes in bowel habits, or rectal bleeding. His last bowel movement was reported the day prior to presentation and was normal in consistency.

On arrival, the patient was afebrile and hemodynamically stable (BP: 126/78 mmHg; HR: 96 bpm; RR: 18; SpO₂: 98%). Abdominal examination revealed mild distension, tenderness in the right and left upper quadrants, and hypoactive bowel sounds. There was no rebound tenderness, guarding, or palpable mass. The remainder of the systemic examination was unremarkable.

Initial laboratory investigations revealed a hemoglobin level of 11.7 g/dL (reference: 13.5-17.5 g/dL), consistent with mild anemia. Although not profound, this finding may reflect chronic blood loss or anemia of inflammation, possibly related to intermittent mucosal irritation or low-grade ischemia secondary to intussusception. The platelet count was elevated at 549 x 10⁹/L (reference: 150-450 x 10⁹/L), suggesting reactive thrombocytosis, which can occur in response to inflammation or tissue stress (Table 1).

**Table 1:** Laboratory results WBC: white blood cell; HGB: hemoglobin; PLT: platelet; K: potassium; PTT: partial thromboplastin time; PT: prothrombin time; INR: international normalized ratio

Test	Result	Reference range
WBC	8.48 x 10^9^/L	4.0-11.0 x 10^9^ /L
HGB	11.7 g/dL	13.5-17.5 g/dL
PLT	549 x 10^9^/L	150-450 x 10^9^/L
K+	5.16 mmol/L	3.5-5.0 mmol/L
Urea	2.5 mmol/L	2.5-6.7 mmol/L
PTT	38.9 sec	25-35 sec
PT	12.1 sec	10-13 sec
INR	1.07	0.9-1.1

Serum potassium was mildly elevated at 5.16 mmol/L (reference: 3.5-5.0 mmol/L), which may represent a pseudohyperkalemia due to platelet activation or could reflect early alterations in bowel perfusion or absorption. Other electrolytes and renal function tests were within normal limits. Coagulation parameters were normal except for a slightly prolonged partial thromboplastin time (PTT) of 38.9 seconds, which was not clinically significant and did not require correction preoperatively.

Of note, the patient had undergone a mini-gastric bypass procedure in 2020 for weight loss, with no reported postoperative complications. He was not on any regular medications and had not used antibiotics or analgesics recently. There was no family history of gastrointestinal malignancies or inflammatory bowel disease. He was a nonsmoker and denied alcohol use or recreational drug consumption. He had no known drug allergies.

Contrast-enhanced computed tomography (CT) of the abdomen revealed a well-circumscribed, fat-density lesion measuring approximately 5 × 9 × 5.1 cm located within the transverse colon and embedded in the bowel wall, consistent with a colonic lipoma. The mass exhibited homogeneous fat attenuation without calcification or septations.

The CT also demonstrated the classic features of intussusception, including a "target sign" on axial images and a "sausage-shaped" soft-tissue mass on coronal views, consistent with a colo-colic (specifically cecocolic) intussusception. These findings are in line with established radiologic criteria for adult intussusception, which include (1) bowel-within-bowel configuration, (2) presence of a lead point, and (3) signs of mesenteric fat and vessels being dragged into the intussuscipiens [1].

**Figure 1:**
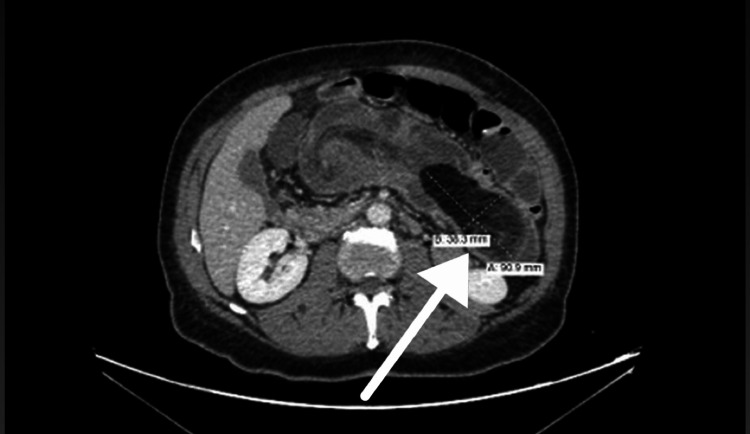
Axial contrast-enhanced CT scan of the abdomen showing a well-defined fat-density lesion in the left upper quadrant, embedded within the large bowel, measuring approximately 5 x 9 x 5.1 cm. The lesion is consistent with a colonic lipoma and serves as the lead point for the intussusception. A red arrow highlights the lipoma

**Figure 2:**
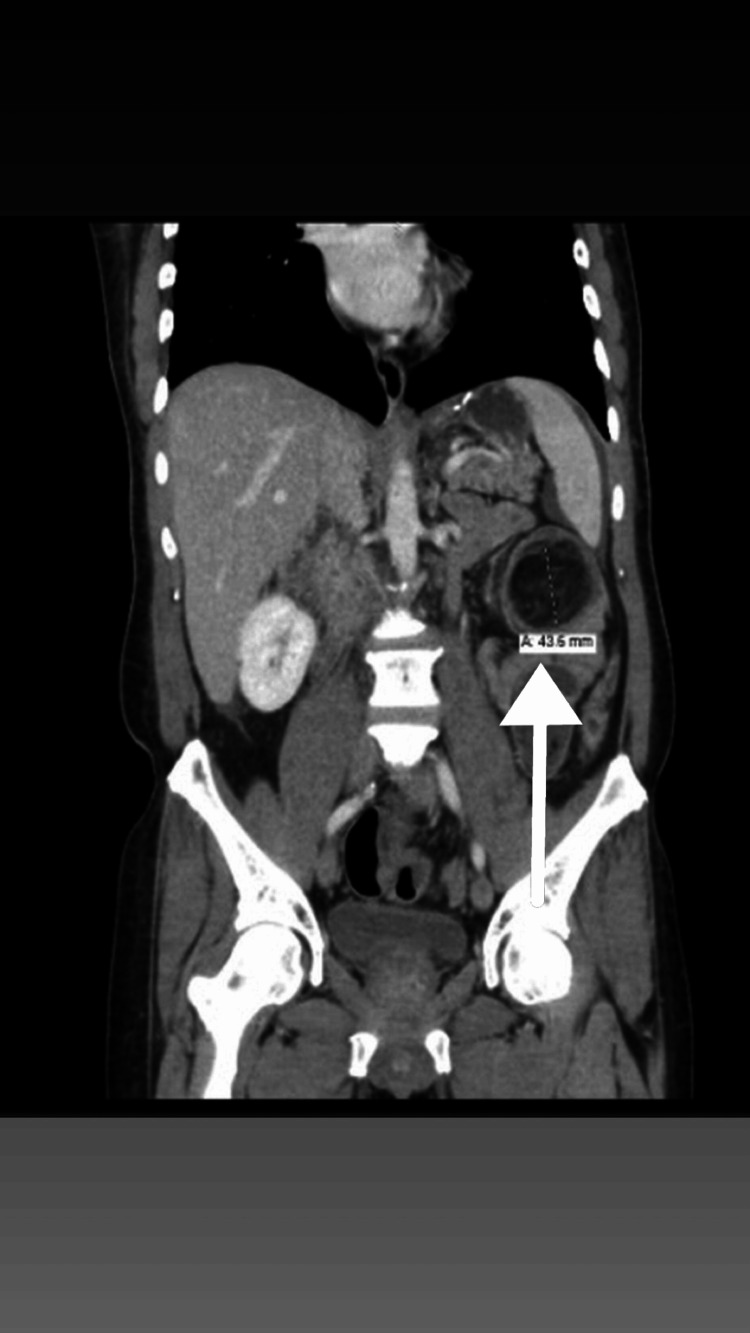
Coronal CT demonstrating cecocolic intussusception Abnormal orientation of the cecum is seen, with concentric alternating layers of bowel and fat directed from the right side of the abdomen toward the left upper quadrant. This image is highly suggestive of cecocolic intussusception (cecum into transverse colon)

**Figure 3:**
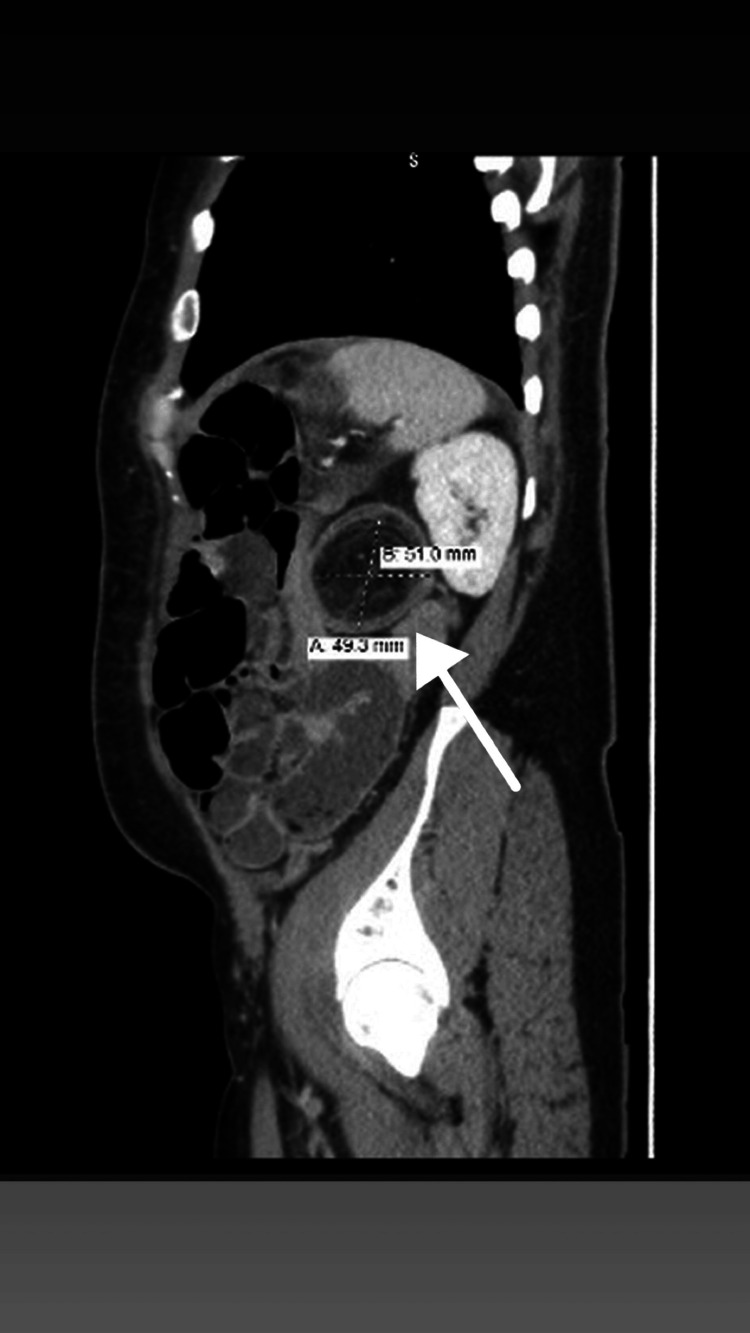
Sagittal CT showing features of partial small bowel obstruction The distal ileum is mildly dilated (up to 3.7 cm) with an element of the small bowel feces sign, suggesting partial obstruction. The invaginated colonic segment demonstrates decreased enhancement as it passes through the intussusception. No pneumatosis is noted

In addition, the distal ileum appeared mildly dilated and demonstrated a “fecalization” sign, indicating delayed transit and partial obstruction. Submucosal edema of the involved colonic segment was noted, but there was no evidence of pneumatosis intestinalis, free air, or signs of bowel perforation (Figures 1-3).

Histopathology results

The patient underwent a limited right hemicolectomy. Gross examination of the resected specimen showed a well-circumscribed, yellow, soft mass located within the submucosal layer of the transverse colon. Histopathological analysis revealed mature adipose tissue arranged in lobules without encapsulation, consistent with a submucosal colonic lipoma. The overlying colonic mucosa appeared intact and unremarkable, with no signs of ulceration, dysplasia, or ischemic change.

Importantly, there were no histologic features suggestive of malignancy. The adipocytes were uniform in size and shape, with no nuclear atypia, lipoblasts, necrosis, or mitotic activity. These findings helped exclude other adipocytic neoplasms such as well-differentiated liposarcoma, which typically show variation in cell morphology, fibrous septa, and lipoblasts.

**Figure 4:**
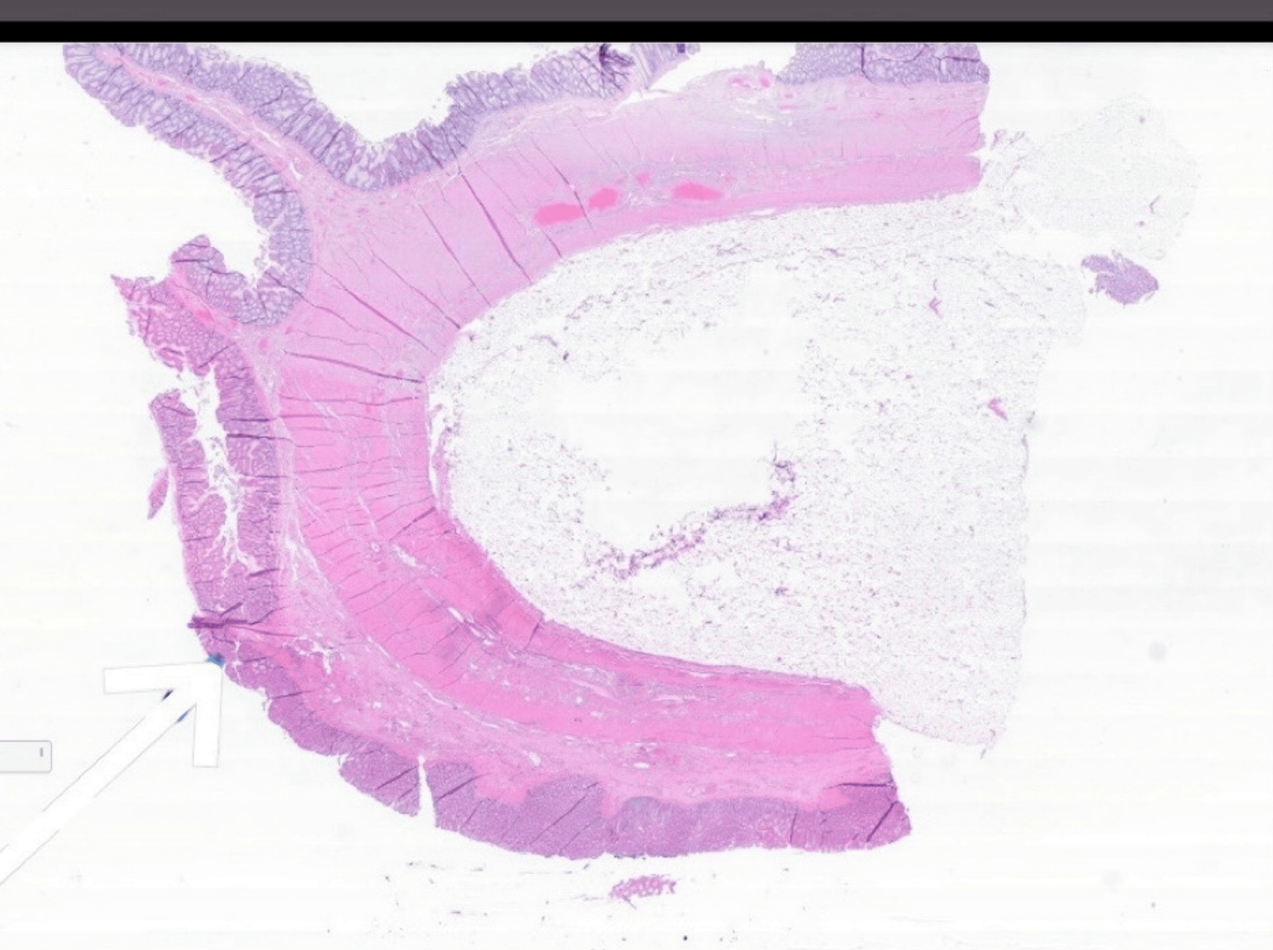
0.4x magnification, H&E stain. This image shows the intact surface of the small intestine with an underlying lipoma. The architecture of the mucosa and submucosal layer is preserved, with the lipoma appearing beneath the normal intestinal structure H&E: hematoxylin and eosin

**Figure 5:**
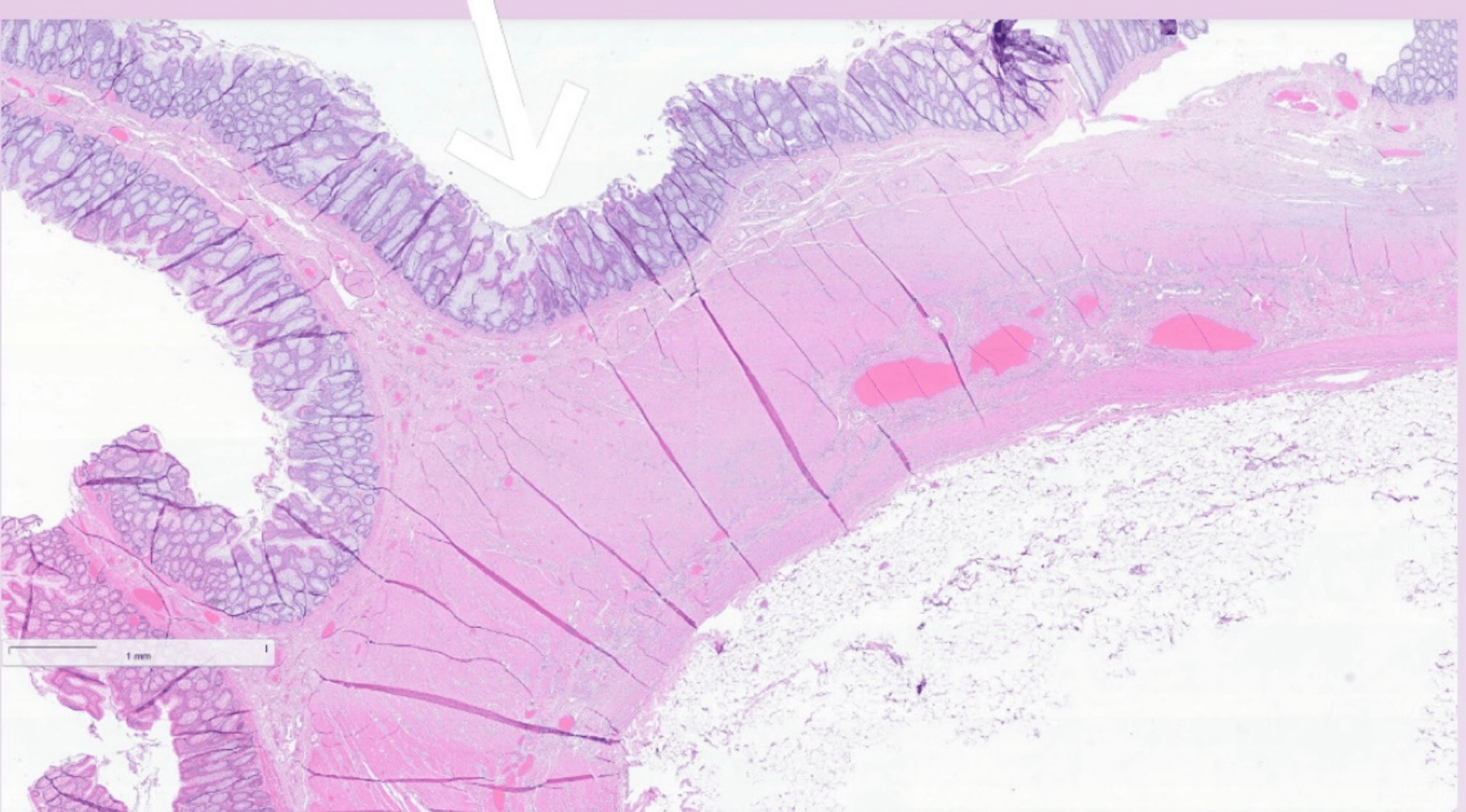
1x magnification, H&E stain. Low-power view showing a submucosal lesion composed of mature adipocytes forming a lobulated mass beneath an intact mucosal surface. The absence of cellular atypia, fibrous septa, or infiltrative margins supports the diagnosis of a benign lipoma H&E: hematoxylin and eosin

**Figure 6:**
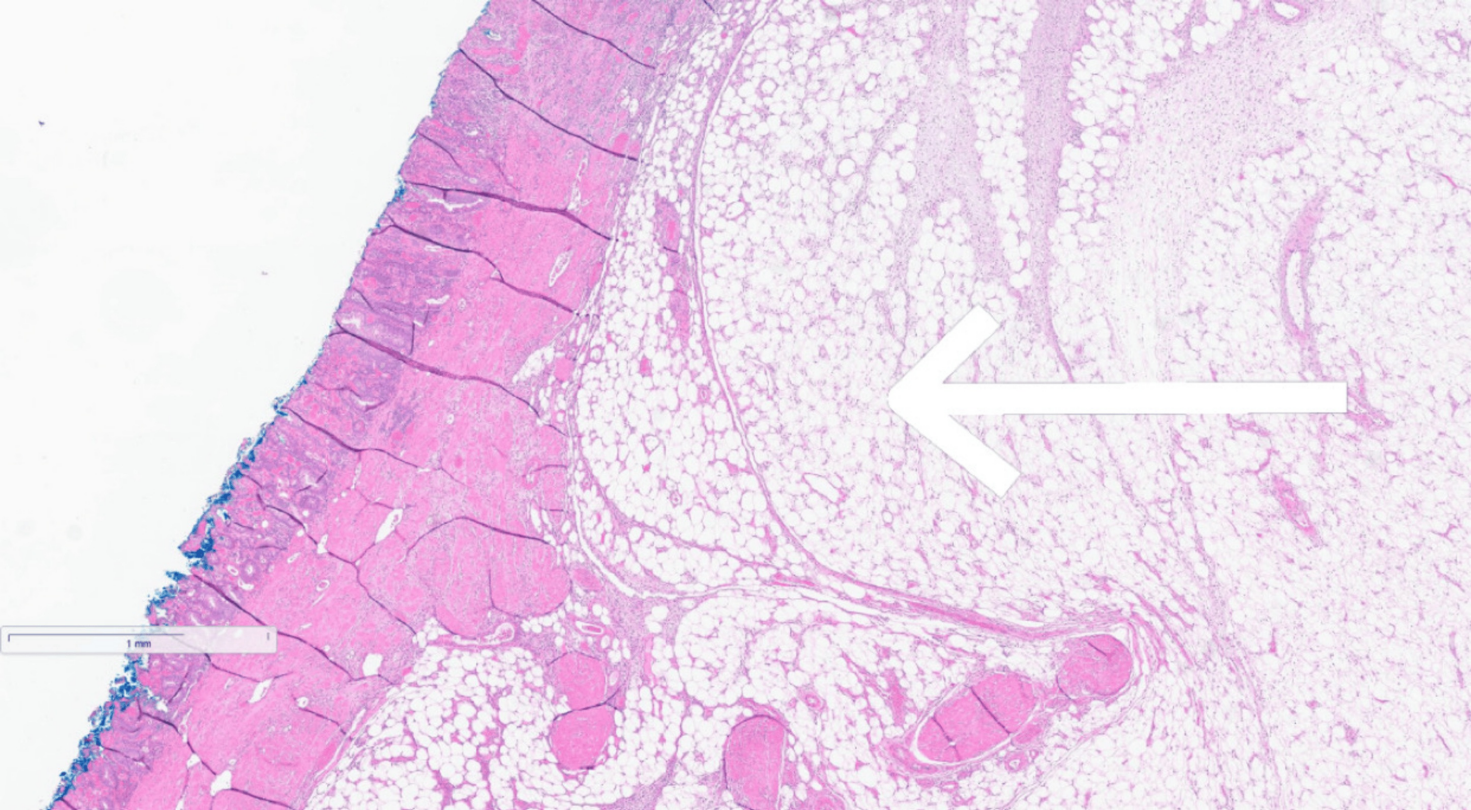
Histologic section highlighting a lobulated mass of mature adipocytes located in the submucosa, with an intact overlying colonic mucosa. The sharp interface between the lipomatous lesion and adjacent tissues, along with the lack of nuclear atypia or infiltrative growth, supports a diagnosis of benign colonic lipoma

The absence of such features confirmed the diagnosis of a benign submucosal lipoma. No other abnormalities or malignancies were identified in the resected tissue (Figures 4-6).

Operative details

An exploratory laparotomy was performed by a senior colorectal surgeon, assisted by a General Surgery Consultant. Intraoperatively, a large, soft, lipomatous-appearing mass was identified within the transverse colon, serving as the lead point for cecocolic intussusception. The intussuscepted segment showed mild congestion but no signs of ischemia or perforation.

**Figure 7:**
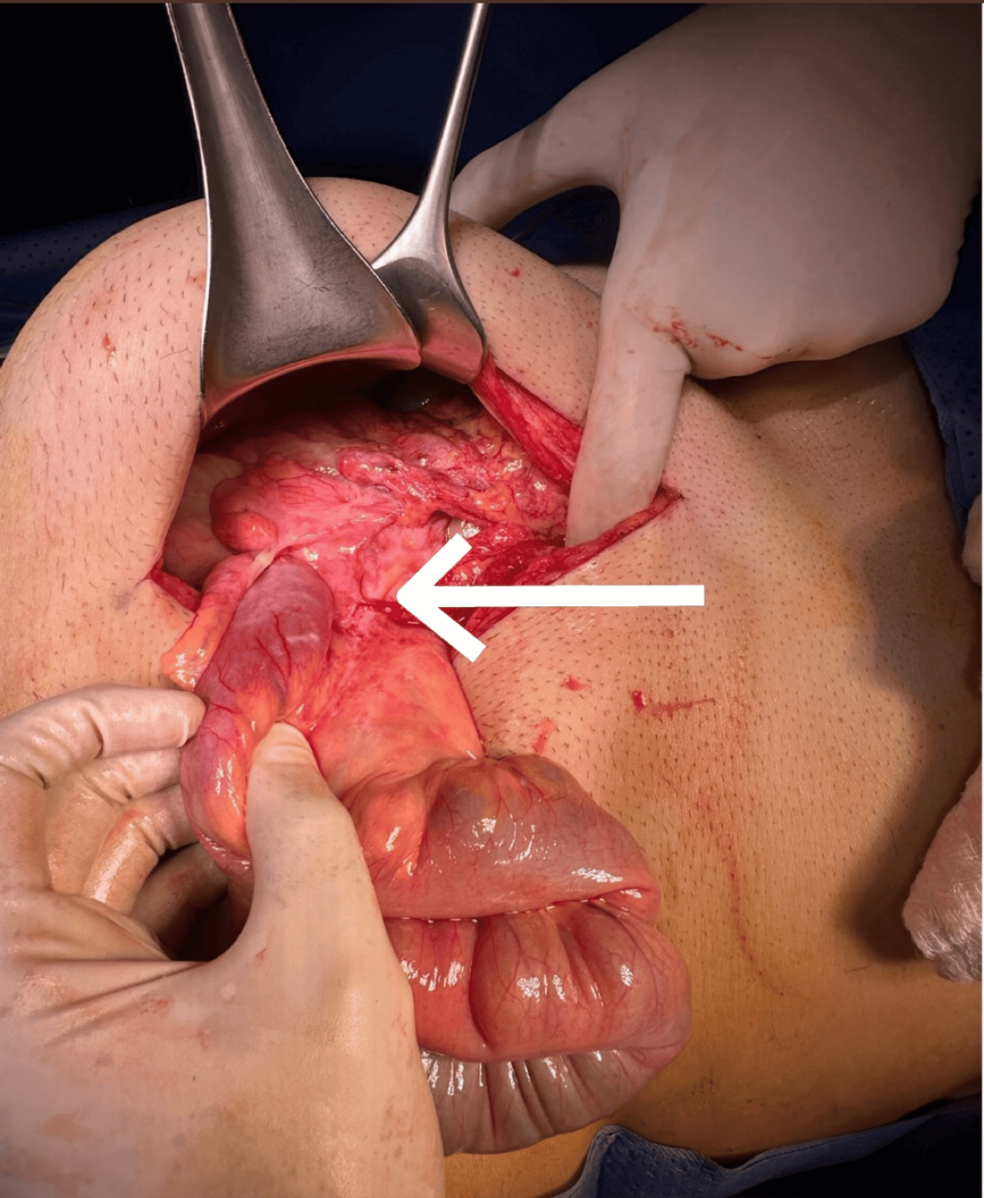
Intraoperative view showing the segment of the colon consistent with cecocolic intussusception

**Figure 8:**
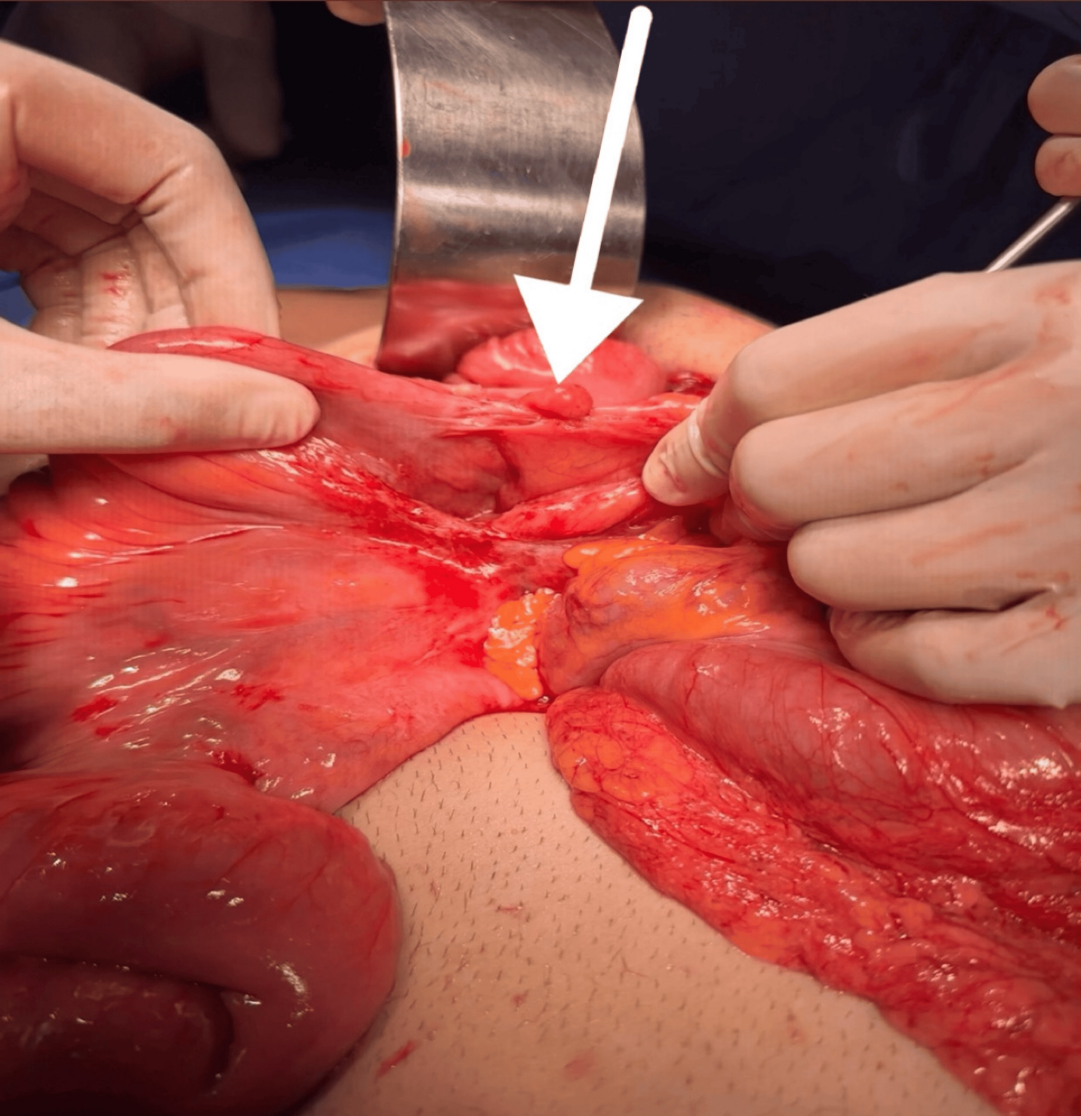
Intraoperative image after gentle reduction of the intussuscepted segment, revealing a well-defined, intraluminal mass palpable within the colonic wall, consistent with a suspected lipoma

**Figure 9:**
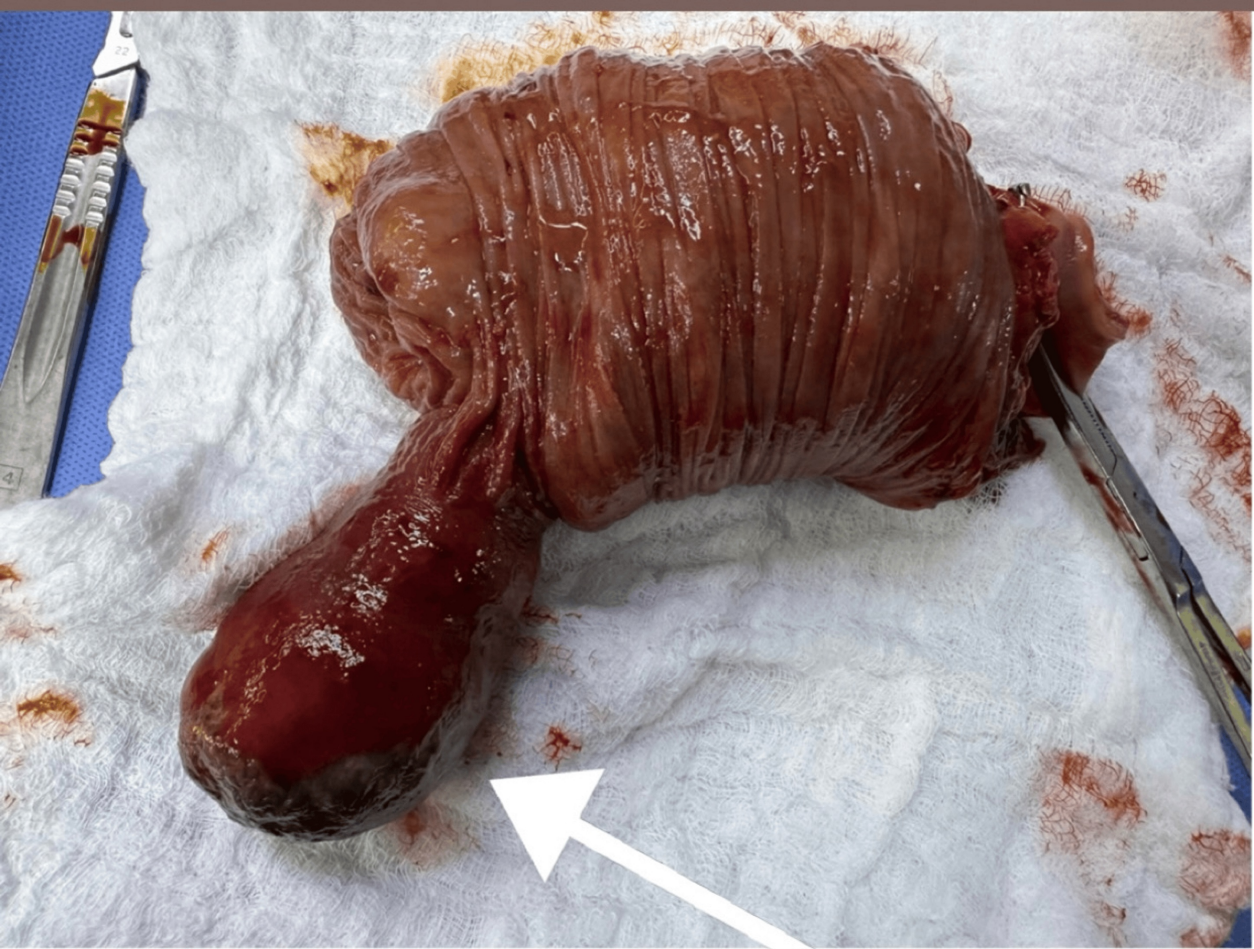
Open view of the colon after removal, demonstrating the submucosal location of the lipoma and the preserved overlying mucosa, confirming a benign intraluminal lesion

Although the lesion was visually suggestive of a benign lipoma, its considerable size and submucosal location raised suspicion for a possible malignant process such as well-differentiated liposarcoma. Given the anatomic inaccessibility of the lesion for endoscopic biopsy due to the invagination, and the limited utility of intraoperative frozen section in differentiating lipoma from liposarcoma, a limited right hemicolectomy with primary ileocolic anastomosis was performed as a definitive and oncologically sound approach (Figures 7-9).

Follow-up and outcome

Postoperatively, the patient had an uneventful recovery. He resumed oral intake on postoperative day 3 and was discharged on day 5. Follow-up at six weeks and three months showed no recurrence of symptoms.

## Discussion

Colonic lipomas, while benign and often incidental, can cause adult intussusception when large enough to act as a mechanical lead point. In our case, contrast-enhanced CT imaging revealed the classic “target sign” and a fat-density lesion within the transverse colon, enabling a preoperative diagnosis of cecocolic intussusception. Importantly, CT excluded perforation or ischemia, which allowed for timely surgical planning [3,4].

The patient's prior mini-gastric bypass surgery presented additional diagnostic complexity. While most cases of postbariatric intussusception involve the small bowel and occur at anastomotic sites, colonic intussusception in this setting is extremely rare. Altered motility, mesenteric laxity, and pressure dynamics after bypass may predispose to abnormal bowel telescoping, even in segments not directly involved in the reconstruction [1,4].

We found no previously published cases describing cecocolic intussusception caused by a lipoma in a patient with prior mini-gastric bypass. Sui et al. reported a similar case of transverse colonic lipoma-induced intussusception, though in a nonbariatric patient [7]. Mehmood et al. described colonic intussusception following colonoscopy, reinforcing the role of lead points and mechanical factors [6]. Menegon Tasselli et al.’s systematic review found that most colonic lipomas leading to intussusception were symptomatic and exceeded 4 cm, with surgical resection commonly performed due to diagnostic uncertainty [8].

Colonoscopy with attempted reduction was not feasible in our case due to the invaginated segment being inaccessible and the lesion’s submucosal characteristics. Additionally, malignancy could not be confidently excluded on imaging or gross inspection [1].

While laparoscopic resection is increasingly utilized for colonic lipomas, we opted for an open approach. Factors influencing this decision included the mass’s size, unclear histologic nature, and the altered anatomy from prior laparoscopic mini-gastric bypass. Although Menegon Tasselli et al. suggest laparoscopy as an option in select cases, an open approach provided better visualization and control [8].

This case emphasizes the importance of cross-sectional imaging in patients with previous abdominal surgery and vague gastrointestinal complaints. It highlights how benign-appearing lesions may still require definitive surgical intervention when anatomical changes and diagnostic ambiguity are present.

## Conclusions

Cecocolic intussusception due to a colonic lipoma is an uncommon condition in adults and may present atypically, especially in patients with prior gastrointestinal surgeries such as mini-gastric bypass. This case highlights the critical role of CT imaging not only in identifying intussusception but also in navigating diagnostic uncertainty in patients with surgically altered anatomy. Surgical resection remains the definitive treatment when malignancy cannot be excluded, particularly for large submucosal lesions inaccessible to endoscopic assessment. Our patient had an uneventful postoperative recovery, with complete resolution of symptoms and return to normal daily activity by the three-month follow-up, underscoring the effectiveness of timely surgical intervention in preserving long-term quality of life.
